# Mating Performance and Singlehood Across 14 Nations

**DOI:** 10.1177/14747049221150169

**Published:** 2023-01-03

**Authors:** Menelaos Apostolou, Mark Sullman, Béla Birkás, Agata Błachnio, Ekaterina Bushina, Fran Calvo, William Costello, Tanja Dujlovic, Tetiana Hill, Timo Juhani Lajunen, Yanina Lisun, Denisse Manrique-Millones, Oscar Manrique-Pino, Norbert Meskó, Martin Nechtelberger, Yohsuke Ohtsubo, Christian Kenji Ollhoff, Aneta Przepiórka, Ádám Putz, Mariaelena Tagliabue, Burcu Tekeş, Andrew Thomas, Jaroslava Varella Valentova, Marco Antonio Correa Varella, Yan Wang, Paula Wright, Sílvia Font-Mayolas

**Affiliations:** 1 Department of Social Sciences, 121343 University of Nicosia, Nicosia, Cyprus; 2 Department of Behavioural Sciences, Medical School, University of Pécs, Pécs, Hungary; 3 Institute of Psychology, The John Paul II Catholic University of Lublin, Lublin, Poland; 4 Center for Sociocultural Research, 68192 National Research University Higher School of Economics, Moscow, Russia; 5 Department of Pedagogy, Quality of Life Research Institute, 16738 Universitat de Girona, Catalonia, Spain; 6 Department of Psychology, The University of Texas at Austin, Austin, USA; 7 Austrian Academy of Psychology (AAP), Vienna, Austria; 8 Business School, 3769 University of Hertfordshire, Hatfield, UK; 9 Department of Psychology, 8018 Norwegian University of Science and Technology (NTNU), Trondheim, Norway; 10 Department of Journalism and Advertising, 187487 Kyiv National University of Trade and Economics, Kyiv, Ukraine; 11 Department of Psychology, 187071 Universidad Científica del Sur, Lima, Peru; 12 251839 Universidad Católica Sedes Sapientiae, Lima, Perú; 13 Institute of Psychology, 37656 University of Pécs, Pécs, Hungary; 14 52732 Carinthia University of Applied Sciences, Villach, Austria; 15 Department of Social Psychology, Graduate School of Humanities and Sociology, 13143 University of Tokyo, Bunkyo-ku, Japan; 16 Department of Experimental Psychology, Institute of Psychology, 28133 University of Sao Paulo, São Paulo, Brazil; 17 Department of General Psychology, 9308 University of Padova, Padova, Italy; 18 Department of Psychology, 589869 Başkent University, Ankara, Turkey; 19 College of Human and Health Sciences, 7759 Swansea University, Swansea, UK; 20 Department of Psychology, 12478 Fudan University, Shanghai, China; 21 3890 Department of Life Sciences, Brunel University London, Uxbridge, UK; 22 Department of Psychology, Quality of Life Research Institute, 16738 Universitat de Girona, Girona, Spain

**Keywords:** mating performance, involuntary singlehood, singlehood, mating, relationship, romantic relationships

## Abstract

Adult individuals frequently face difficulties in attracting and keeping mates, which is an important driver of singlehood. In the current research, we investigated the mating performance (i.e., how well people do in attracting and retaining intimate partners) and singlehood status in 14 different countries, namely Austria, Brazil, China, Greece, Hungary, Italy, Japan, Peru, Poland, Russia, Spain, Turkey, the UK, and Ukraine (*N* = 7,181). We found that poor mating performance was in high occurrence, with about one in four participants scoring low in this dimension, and more than 57% facing difficulties in starting and/or keeping a relationship. Men and women did not differ in their mating performance scores, but there was a small yet significant effect of age, with older participants indicating higher mating performance. Moreover, nearly 13% of the participants indicated that they were involuntarily single, which accounted for about one-third of the singles in the sample. In addition, more than 15% of the participants indicated that they were voluntarily single, and 10% were between-relationships single. We also found that poor mating performance was associated with an increased likelihood of voluntary, involuntary, and between-relationships singlehood. All types of singlehood were in higher occurrence in younger participants. Although there was some cross-cultural variation, the results were generally consistent across samples.

## Introduction

Attracting and keeping an intimate partner is a challenging endeavor that is fraught with difficulties (Apostolou et al., 2018; Cherlin, 2009). Yet, there has been limited research on mating performance, so we know little about how widespread the phenomenon is. Difficulties dealing with the challenges of mating are associated with an increased likelihood of being involuntarily single, that is, not being in an intimate relationship although one wishes to be so (Apostolou & Wang, 2019). People may also choose to be single to work on developing their strengths or become temporarily single when their relationship ends (Apostolou, 2017). Although singlehood appears to be a common state in contemporary societies, there is surprising little research in the area. Actually, most scholars use the term single to mean not married rather than not in an intimate relationship (see Klinenberg, 2012). Consequently, little is known about the occurrence of the different types of singlehood, as well as their association with poor mating performance. The purpose of the current work is to address these gaps in the literature by examining mating performance, singlehood status, and the association between the two in a sample from 14 different nations. Difficulties in mating and singlehood can be understood within an evolutionary theoretical framework that will be discussed next.

### The Evolutionary Roots of Poor Mating Performance

As all traits exhibit variation (Fisher, 1958; see also Buss, 2009), so does mating performance; that is, some do better than others in attracting and retaining intimate partners (Apostolou et al., 2018). One reason is genetic mutations, which can affect the development of mechanisms involved in mating, impairing their functioning (Keller & Miller, 2006). Similarly, environmental stressors such as been exposed to harmful substances can also affect the development and functioning of these mechanisms. Given that the DNA replicates in a reliable fashion, and making the reasonable assumption that people have evolved some resistance to environmental stressors, these factors would predict the presence of only a small variation between individuals in mating performance. Yet, the observed variation is considerable. For instance, a study measuring mating performance in China and Greece found that about half of the participants reported facing difficulties in attracting and retaining mates (Apostolou & Wang, 2019). The observed high level of variation is evolutionary puzzling, as poor mating performance may have high reproductive or fitness costs. This means that those who do poorly in attracting and retaining intimate partners have fewer offspring than those who do better in these areas.

It has been argued that this paradox could be partially resolved, by taking into consideration the fact that ancestral mating conditions were considerably different from the contemporary ones (Apostolou, 2015). More specifically, adaptations are mechanisms that have evolved to solve the problems of survival and reproduction that our ancestors faced (Buss, 2020). However, these adaptations have evolved to solve these problems in an ancestral context, and so, they may not be as effective in the modern environment. This is known as the mismatch problem (Crawford, 1998; Goetz et al., 2019; Li et al., 2018) and can potentially explain the observed variations in mating performance.

There are various lines of evidence to suggest that the ancestral mating market was very different from the contemporary one. To begin with, anthropological evidence indicates that in contemporary preindustrial societies (i.e., societies in which most people base their subsistence on hunting and gathering, or farming and animal herding), the typical form of long-term mating is via an arranged marriage (Apostolou, 2014; Broude & Green, 1983). For instance, one study examined evidence from 190 contemporary hunting and gathering societies and found that in about 70% of the cases marriages were arranged, and only in about 4% of the cases, individuals were free to choose their own spouses (Apostolou, 2007). A subsequent study found that arranged marriage was also the most prevalent mode of long-term mating in societies which based their subsistence on agriculture and/or animal herding (Apostolou, 2010). As the way of life in contemporary preindustrial societies is likely to be similar to ancestral societies (Lee & Devore, 1968, but see Ember, 1978), it is reasonable to assume that arranged marriage was also prevalent in ancestral human societies. Phylogenetic (Walker et al., 2011) and historical evidence (Apostolou, 2012; Coontz, 2006) corroborates this prediction. Furthermore, men form male alliances to fight other men and gain access to their resources, as well as to women (Tooby & Cosmides, 1988). Anthropological, historical, archeological and physiological evidence indicates that such instances were relatively common in ancestral human societies (Lidborg et al., 2022; Puts, 2010, 2016).

People in post-industrial societies (i.e., societies in which most people are employed in the services sector) have to solve the same evolutionary problem our ancestors faced, namely how to attract and retain mates. To do so, they need to rely on adaptations, which have evolved to solve this problem in a context where mate choice was predominantly regulated or forced. Even if the evolutionary problem is the same, these adaptations may not be effective because the different mating environment requires different ways to solve it. To use one example, people today would look for mates online (Dinh et al., 2021), which could potentially give them hundreds or even thousands of options. Such a plethora of options may tax individuals’ mental hardware, which has not evolved to deal with so many choices, resulting in people doing poorly in finding mates online (Thomas et al., 2022; for further discussion on mating and reproductive success in contemporary societies, see Gutiérrez et al., 2022; Međedović, 2021, 2022).

Natural selection would act on adaptations involved in mating adjusting them to the contemporary context, but this process requires considerable time. The transition to post-industrialism started with the industrial revolution in the 18th century, allowing very little time, in evolutionary terms, for such an adjustment to take place. Accordingly, we would expect that several people today have adaptations that are not able to solve effectively problems associated with mating, and consequently, they experience poor mating performance (Apostolou, 2015; Goetz et al., 2019). To use one example, in the ancestral context where individual rights were not well protected and access to women could be monopolized by force, high male aggression would be selected, as it would enable men to monopolize access to women as well as guarding their partners (Buss, 2021). High male aggression is likely to have the opposite effect in a post-industrial context, where individual rights are well protected, and mate choice is freely exercised and not forced. This argument leads to the prediction that, across post-industrial societies, a considerable proportion of the population experiences poor mating performance. Currently, there is only evidence that this is the case in China and Greece, which is insufficient for testing this prediction. Accordingly, the current study aimed to examine this phenomenon in a larger sample of post-industrial societies.

### Involuntary, Voluntary, and Between-Relationships Singlehood

If people experience poor mating performance, that is, they face difficulties in attracting and retaining mates, they would be more likely to be single without wanting to be so, in comparison to people who do well in this domain. Apostolou and Wang (2019) tested this prediction in Chinese and Greek samples and found that a decrease in mating performance was associated with a considerable increase in the probability of being involuntarily single. The current study aimed to extend this work by examining whether this prediction holds in other sociocultural and geographic contexts.

Moreover, people who experience poor mating performance are likely to encounter repeated failures in their attempts to attract and retain mates, so eventually, they may give up, preferring to be single instead. Accordingly, it is predicted that poor mating performance would be associated with an increased likelihood to prefer to be single, a prediction that the current research aimed to test. Assuming that poor mating performance is relatively common, and it is also associated with an increased probability of being involuntarily and voluntarily single, then involuntary and voluntary singlehood would also be common, a prediction that the present study aimed to test.

Post-industrial societies are technology-oriented, which means that individuals living in them need to spend considerable time in training prior to participating effectively in the labor force. Successful participation in the labor force is associated with the capacity to provide resources to one's family, which, in turn, is highly valued in the mating market (Buss, 2016). Accordingly, it has been argued that staying single for some time could be beneficial for people, as it allows them to focus on developing their strengths that, in turn, would enable them to attract better value mates at a future time (Apostolou, 2017). Thus, another reason why voluntary singlehood is predicted to be common in post-industrial societies is that people would opt out of the mating market to divert the bulk of their resources to advancing their studies and careers. This argument is supported by the results of a cross-cultural study employing a sample representing eight different post-industrial societies, where participants reported that they were single to focus on their careers (Apostolou et al., 2022).

Moving on, it has been further argued that single life could be better than mated life, as the former is associated with more positive outcomes than the latter (DePaulo, 2007; Trimberger, 2006). For example, singles have more time to develop themselves (DePaulo, 2007), allocate more time to do physical exercise and can potentially be healthier (Nomaguchi & Bianchi, 2004), have more friends (Adams, 1976; Sarkisian & Gerstel, 2016), and spend more time with their relatives (Sarkisian & Gerstel, 2016). The possible benefits of singlehood may make it more desirable for some people, who may prefer to stay single.

Furthermore, people's intimate partners may die in an accident, fight, or from a serious illness or abandon them for other mates. One reason for the latter is that they may come to realize that their current mates are not appropriate for them, so they will terminate the relationship and look for partners who are a better fit. This is likely to be a common occurrence, due to deception and the fact that certain traits, such as kindness, are difficult to assess in a potential partner (Buss, 2017; Toma & Hancock, 2010; see also Miller, 2000). Another reason is the mismatch problem. More specifically, in ancestral human societies, screening was to some degree made by parents, so individuals’ screening mechanisms may not be sufficient for the demands of a context where people have to choose their own mates (Apostolou, 2014). Therefore, people would frequently find themselves in relationships with partners who are not a good fit and so would become motivated to terminate their relationship and to look for a better partner. Overall, inept evaluation skills may lead people into many short-term relationships. For instance, one qualitative study found the “Attracted to wrong women” and the “Bad experiences from previous relationships” to be common reasons for being single (Apostolou, 2019). In effect, at any given point in time, many people would be between relationships, since they have recently exited a relationship and are single for a short period before entering another. The present study aimed to examine this prediction.

### The Current Study

Many people face difficulties in mating and are willingly or unwillingly single. However, there is surprisingly little research in the area, and attempts to study these phenomena have been limited to the Chinese and the Greek contexts. The current study aimed to close the gap in our knowledge by testing three hypotheses in a sample from 14 nations. More specifically, the theoretical framework discussed above predicts that a sizable proportion of people living in post-industrial societies would face difficulties in attracting and retaining intimate partners (H_1_) and that poor mating performance would be associated with an elevated probability to be voluntarily and involuntarily single (H_2_). With respect to the latter hypothesis, it could be argued that it does not require testing as the relationship between the two phenomena is apparent. Yet, there are at least two reasons that this hypothesis needs to be tested. First, an apparent relationship does not guarantee an actual relationship between two phenomena. For example, it could be the case that those who are not good in intimate relationships (e.g., have poor flirting skill) exercise considerable more mating effort than those who are good in it, and this extra effort balances their shortcoming, so they are not more likely to be single than the latter. Second, poor mating performance may be associated with more failures in mating, but these failures may not necessarily lead people to give up on looking for mates and prefer instead to be single.

Finally, this framework predicts that the three main types of singlehood (i.e., voluntary, involuntary, and between relationships) would be relatively common (H_3_).

## Methods

### Participants

The data presented in the current research was a part of a larger study on human mating. In total, 7,181 (4,616 women, 2,525 men, and 40 participants who did not indicate their sex) individuals took part. Participants were recruited from 14 different countries, namely, Austria, Brazil, China, Greece, Hungary, Italy, Japan, Peru, Poland, Russia, Spain, Turkey, the UK, and Ukraine. The number of participants for each country, along with their mean age, is presented in Table 1. Moreover, we sampled from both Greece and the Republic of Cyprus, and in both cases, the responses came from a Greek population, so we treated the sample as one (i.e., Greece). The study received ethics approval from the respective ethics committees in each country. The only requirement for participation was to be at least 18 years old.

**Table 1.** Number of Participants and Age Across the 14 Samples.

				Mean age (SD)	
Countries	*N*	Women	Men	Women	Men
Total	7,141^a^	4,616	2,525	31.4 (15.4)	35.9 (15.4)
Austria	373	295	74	35.1 (9.8)	40.3 (14.1)
Brazil	648	453	183	32.7 (13.3)	30.4 (12.7)
China	537	278	259	26.5 (3.3)	26.6 (3.6)
Greece	566	366	199	29.5 (10.4)	29.4 (10.8)
Hungary	500	401	95	28.6 (10.3)	30.5 (12.8)
Italy	468	323	141	29.3 (10.5)	35.1 (10.5)
Japan	706	338	365	48.4 (13.0)	56.3 (11.0)
Peru	640	356	284	21.4 (5.0)	22.1 (5.1)
Poland	513	261	252	41.7 (12.2)	47.4 (13.9)
Russia	402	285	116	39.2 (10.3)	42.4 (9.8)
Spain	356	274	78	28.0 (10.7)	34.6 (12.8)
Turkey	953	684	263	25.4 (9.0)	25.9 (9.3)
UK	360	215	144	28.5 (10.8)	35.5 (13.9)
Ukraine	159	87	72	37.4 (12.0)	44.2 (9.2)

^a^ There were also 40 participants who did not indicate their sex.

For the Japanese and the Polish samples, participants received monetary compensation or credits that could be exchanged for a product. For the UK, Ukrainian, and Russian samples, participation was both on a voluntary and on a compensation basis. For the rest of the samples, participation was on a voluntary basis only. Japanese participants were recruited using a private recruitment agency (https://www.cross-m.co.jp/). Similarly, a private agency was also used for recruiting participants from Russia and Ukraine (anketolog.ru). For the UK sample, some of the participants were recruited using the Prolific platform, while Polish participants were recruited from a Polish national survey panel (http://panelariadna.pl/). For the rest of the samples, participants were recruited by promoting the link to the study through social media, and by forwarding it to students and colleagues.

### Materials

The instruments were translated into the primary language of each country in the sample. The survey was conducted online and was created using the Google Forms, Microsoft Forms, Qualtrics, or Sojump tools. To measure mating performance, we employed a five-item instrument developed by Apostolou et al. (2018). The respective items of the instrument were listed in Table 2. Previous studies have found that this instrument had criterion validity; in that, it was associated with flirting skills, mate signal-detection ability, shyness, emotional intelligence, length of singlehood spells, sexual functioning, and mating effort (Apostolou et al., 2018, 2019a, 2019b; Apostolou & Wang, 2019).

**Table 2.** Frequencies of Participants’ Responses in the Mating Performance Instrument.

	I find romantic relationships difficult			I do well in romantic relationships			I find it easy to start a romantic relationship			I find it easy to keep a romantic relationship			Some people are doing well with romantic relationships. They find partners easily and have no difficulty in keeping a romantic relationship. This description characterizes me			
Countries	1–2	3	**4–5**	**1–2**	3	4–5	**1–2**	3	4–5	**1–2**	3	4–5	**1–2**	3	4–5	Performance^a^
Total	36.2	25.4	**38.4**	**32.6**	28.7	38.7	**48.2**	23.3	28.6	**38.4**	28.0	33.6	**45.6**	25.6	28.8	57.5
Austria	53.1	23.9	**23.0**	**19.7**	30.2	50.2	**41.1**	23.1	25.8	**24.1**	25.5	50.4	**34.7**	26.3	39.0	45.7
Brazil	25.7	20.6	**53.8**	**34.0**	25.5	40.6	**60.7**	14.1	25.2	**52.0**	23.9	24.0	**58.2**	20.1	21.7	74.4
China	31.8	23.3	**44.8**	**7.4**	12.8	78.4	**19.2**	22.7	58.1	**18.2**	18.6	63.2	**11.9**	16.4	71.7	24.2
Greece	48.9	21.2	**29.9**	**28.8**	28.3	42.9	**38.2**	22.6	39.3	**24.9**	28.1	47.0	**43.7**	26.0	30.3	44.4
Hungary	50.4	18.0	**31.6**	**50.2**	17.6	32.2	**24.6**	22.4	53.0	**45.8**	20.7	33.6	**28.8**	24.2	47.0	47.4
Italy	41.7	24.1	**24.1**	**36.3**	31	32.7	**55.6**	18.8	25.6	**38.2**	31.6	30.2	**56.8**	22.0	21.2	66.5
Japan	14.9	27.2	**57.9**	**67.3**	22.1	10.7	**68.8**	22.7	8.5	**65.9**	26.8	7.4	**69.0**	24.5	6.5	79.3
Peru	35.9	34.4	**29.7**	**20.6**	37.8	41.6	**42.3**	33.1	24.5	**29.2**	34.2	36.5	**38.6**	32.0	29.3	52.0
Poland	46.4	31.4	**22.2**	**26.3**	36.1	37.7	**33.7**	36.5	29.8	**28.7**	36.1	35.3	**34.7**	34.5	30.8	38.4
Russia	47.8	29.1	**23.1**	**43.0**	34.3	22.7	**60.4**	24.4	15.2	**32.3**	33.6	34.1	**53.2**	27.4	19.4	64.7
Spain	33.9	21.8	**44.4**	**31.1**	29.1	39.8	**57.1**	17.2	25.7	**35.1**	26.1	38.8	**55.4**	18.6	26.0	64.6
Turkey	23.9	23.6	**52.5**	**24.9**	31.5	43.7	**58.9**	21.3	19.8	**47.7**	28.1	24.1	**50.7**	26.0	23.3	70.2
UK	43.1	26.7	**30.2**	**27.2**	34.2	38.6	**52.5**	20.8	26.7	**–**	–	–	**49.7**	25.8	24.4	–
Ukraine	28.9	42.8	**12.5**	**33.3**	46.5	20.1	**52.8**	28.9	18.3	**22.6**	40.3	37.1	**37.7**	50.3	11.9	56.6

*Note*. The numbers above reflect the percentages of participants’ answers in each question of the instrument, which employed the scale 1—strongly disagree, 5—strongly agree. The percentages that indicate poor mating performance are in bold.

^a^ In this column, we have calculated the percentage of participants who have scored “1” and “2” in either or both the questions related to how easy they found to start and keep an intimate relationship.

Participants’ responses were recorded using a five-point Likert scale, which ranged from 1—Strongly disagree to 5—Strongly agree, with a higher total score indicating higher mating performance. To measure relationship status, we employed an instrument with the following categories: “Between-relationships single: My relationship has recently ended and I have not yet found another partner,” “Voluntarily single: I am not interested in being in a relationship,” “Involuntarily single: I want to be in a relationship, but I find it difficult to attract a mate,” “In a relationship,” “Married,” and “Other” (Apostolou & Wang, 2019). In eight samples (Table G), participants were also asked to indicate for how many years they had been single (see Supplementary Material G for further analysis).

### Data Analysis

To test the hypothesis that poor mating performance would be in high occurrence (H_1_), we calculated the mean scores of mating performance as well as the frequencies of participants’ answers in the different items of the mating performance instrument. Furthermore, to estimate sex, age, and sample effects on mating performance, we run an ANCOVA test, where mating performance was entered as the dependent variable, sex and the sample (14 levels, one for each country) were entered as the categorical independent factors, and age as the continuous independent factor. Pre-test examination of the data indicated that the normality assumption held, the slopes of the regression lines were generally homogenous, with the exception of Chinese sample, which had a steeper regression line than the rest. In addition, there was a moderate violation of the homogeneity of variance assumption. However, the ANCOVA test is robust to violation of its assumptions, especially when the sample is large (Olejnik & Algina, 1984).

To test the hypothesis that the three types of singlehood would be relatively common (H_3_), we calculated their respective frequencies for the pooled, as well as the individual country samples. Moreover, to examine whether mating performance predicted singlehood status (H_2_), we performed a series of multinomial regressions where relationship status was entered as the dependent variable, and the mean mating performance scores, sex, sample and age were entered as independent variables. For some samples, there were very few observations in the “other” relationship status category, so the model could not be fitted. Accordingly, in our analysis we dropped the “other” category.

## Results

### Mating Performance (H_1_)

As seen in Table 2, about one-third of the participants found intimate relationships difficult, nearly half found it difficult to start a relationship, and 38% found it difficult to maintain a relationship. Furthermore, there was substantial cross-cultural variation. About 19% of the Chinese participants indicated that they faced difficulties starting an intimate relationship, compared to more than 60% of Japanese participants. Yet, in most cases, the percentage was above 40, indicating that starting an intimate relationship was a problem in most countries in our sample. Furthermore, we calculated that more than 57% of the participants indicated that they faced difficulties in starting and/or keeping a relationship. Although there was variation across the samples, in most cases the percentage was above 50, indicating that the majority of people across these countries faced difficulties in at least one aspect of intimate relationships. We also calculated the mean mating performance score for each sample. As we can see from Table 3, the highest mean was for China and the lowest for Japan. In addition, we calculated the percentage of participants who indicated a low mating performance (i.e., a mean score of two or less). This shows that about one in four participants indicated low mating performance. The frequencies in Table 2 suggest that it was more difficult for people to start than to keep an intimate relationship, a difference that subsequent analysis found to be significant (Supplementary Material A).

**Table 3.** Sex, Age, and Sample Differences in Mating Performance.

				Sex				Age^a^		Sample^b^
Countries	Mean (SD)	% equal or below 2	Rank	Women	Men	*p*-Value	*η_p_* ^2^	*p*-Value	*η_p_* ^2^	*p*-Value^c^
Total	2.86 (1.01)	24.8		2.86 (1.01)	2.87 (0.99)	.359	.000	<.001	.004	<.001
Austria	3.26 (0.99)	17.6	2	3.27 (1)	3.21 (0.90)	.533	.001	.466	.002	T,C,B,I,R,S,J,UK
Brazil	2.56 (1.04)	37.5	13	2.55 (1.06)	2.59 (1.02)	.539	.001	.033	.007	G,T,A,P,H,J,Pe,UK
China	3.58 (0.77)	5.8	1	3.53 (0.84)	3.64 (0.68)	.101	.005	<.001	.060	G,T,A,B,I,R,U,P,S,H,J,Pe,UK
Greece	3.13 (1.05)	18.1	3	3.14 (1)	3.09 (1.09)	.624	.000	.065	.006	T,C,B,I,R,S,J,UK
Hungary	3.11 (1.12)	22.0	4	3.15 (1.11)	2.95 (1.12)	.064	.007	<.001	.025	T,C,B,I,R,S,J
Italy	2.77 (0.98)	26.3	10	2.77 (1.02)	2.78 (0.89)	.675	.000	.022	.011	G,C,A,P,H,J,Pe
Japan	2.07 (0.81)	53.7	14	2.06 (0.80)	2.09 (0.82)	.737	.000	.536	.001	G,T,A,B,I,R,U,P,S,H,J,Pe,UK
Peru	2.99 (0.85)	14.2	6	2.89 (0.86)	3.12 (0.83)	.001	.016	.002	.015	T,C,B,I,R,S,J
Poland	3.08 (0.95)	16.2	5	3.12 (0.99)	3.03 (0.91)	.036	.009	.539	.001	T,C,B,I,R,S,J
Russia	2.74 (0.93)	26.1	11	2.75 (0.93)	2.75 (0.96)	.970	.000	.998	.000	G,C,A,P,H,J,Pe
Spain	2.79 (1.02)	28.6	9	2.79 (1.03)	2.78 (0.98)	.571	.001	.050	.011	G,C,A,P,H,J,Pe
Turkey	2.71 (0.85)	25.4	12	2.64 (0.82)	2.91 (0.90)	<.001	.020	.072	.003	G,C,A,B,P,H,J,Pe
UK	2.87 (1.00)	24.4	8	2.97 (1.00)	2.73 (0.99)	.020	.015	.448	.002	G,V,A, B,J
Ukraine	2.89 (0.65)	8.2	7	2.94 (0.67)	2.82 (0.62)	.253	.008	.979	.000	C,J

^a^ In all instances where age was significant, the regression coefficient was positive.

^b^ In this column, the results of the Bonferroni post hoc test are presented. For each country in the raw, the initials of the countries for which there was a significant difference in the mean mating performance scores are presented. For example, for the first raw, the “T” indicates that Austria was significantly different from Turkey. Note also that “P” refers to Poland and “Pe” to Peru.

^c^ The *η_p_*^2^ for the sample variable was .121.

With respect to the ANCOVA analysis, as we can see from Table 3, there was no significant main effect of sex, but there was a significant main effect of age, with a positive coefficient, suggesting that older participants reported higher mating performance. The effect size indicated that this effect was small. Moreover, there was a significant main effect of sample, which was also small. Post hoc Bonferroni analyses indicated several differences in the mean mating performance scores across the samples. Additionally, we found a significant interaction between sample and sex (*F*(1, 7029) = 3.29, *p *< .001, *η_p_*^2^ = .006), indicating that the effect of sex on mating performance differed across samples. However, the effect size indicated that this variation was very small. We also performed a series of ANCOVA tests using sex and age as independent variables for each individual sample. To avoid the problem of alpha inflation arising from multiple comparisons, Bonferroni correction was applied, reducing the alpha level to .004 (.05/14). Thus, the reader may consider any effect above this level not to be significant. We can see from Table 3 that in most cases there were no significant main effects of sex and age.

### Singlehood Status (H_3_)

Table 4 shows that 38.1% of the participants indicated that they were single, with 12.9% indicating that they were involuntarily and 15.2% voluntarily so. There was considerable variation across samples. For instance, 4.9% of the Polish sample were involuntarily single, compared to 21.8% in the sample from Brazil. To examine whether sample differences in the relationship status were significant, we employed the Chi-squared test of independence. For most cases, the results indicated significant differences between the samples (Table E, Supplementary Material).

**Table 4.** Relationship Status Across Samples.

		Relationship status					
Countries	*N*	Between relationships single	Voluntarily single	Involuntarily single	In a relationship	Married	Other
Total	7161	10.0	15.2	12.9	31.7	26.7	3.4
Austria	373	7.8	2.9	9.7	49.3	27.6	2.7
Brazil	646	6.8	20.6	21.8	29.7	17.3	3.7
China	537	27.2	11.2	11.4	34.5	15.5	0.4
Greece	563	14.2	10.5	19.0	34.6	15.3	6.4
Hungary	500	7.6	8.6	20.8	41.8	17.6	3.6
Italy	468	7.9	7.7	12.2	46.8	20.7	4.7
Japan	706	4.0	12.0	9.9	3.7	64.9	5.5
Peru	626	13.9	41.1	6.1	35.5	3.4	0.2
Poland	513	6.6	6.6	4.9	23.4	55.9	2.5
Russia	402	6.2	7.7	9.7	16.4	58.2	1.7
Spain	355	8.2	18.3	12.7	37.5	17.7	5.6
Turkey	953	8.1	22.2	14.6	36.6	14.9	3.6
UK	360	11.1	15.0	12.2	42.8	14.4	4.4
Ukraine	159	15.1	5.7	10.7	10.1	56.0	2.5

Relationship status is likely to vary considerably with age (e.g., older people are more likely to be married than younger ones). Thus, we calculated the relationship status separately for participants 18–27, 28–37, and ≥38 years of age (Tables B–D in the Supplementary Materials section). We can see that all three types of singlehood were higher in the younger, than in the older age groups. For instance, involuntary singlehood was higher in the younger age group, with nearly 17% of the participants falling in this category, compared to about 11% in the 28–37 age group, and about 8% in the ≥38 group. To examine whether the differences in the frequencies were significant, we performed a Chi-squared test of independence. As we can see in Table F (Supplementary Material), the rate of involuntary singlehood was significantly different across age groups. Furthermore, in Table 5, we calculated the proportions of each type of singlehood. The highest proportion was preferred singlehood (about 40% of the cases), followed by involuntary singlehood (about 34%) and between-relationships singlehood (about 26%).

**Table 5.** Composition of the Singlehood Status.

		Singlehood status		
Countries	*N*	Between relationships single	Voluntarily single	Involuntarily single
Total	2730	26.3	39.9	33.8
Austria	76	38.2	14.5	47.4
Brazil	318	13.8	41.8	44.3
China	267	54.7	22.5	22.8
Greece	246	32.5	24.0	43.5
Hungary	185	20.5	23.2	56.2
Italy	130	28.5	27.7	43.8
Japan	183	15.3	46.4	38.3
Peru	382	22.8	67.3	9.9
Poland	93	36.6	36.6	26.9
Russia	95	26.3	32.6	41.1
Spain	139	20.9	46.8	32.4
Turkey	428	18.0	49.5	32.5
UK	138	29.0	39.1	31.9
Ukraine	50	48.0	18.0	34.0

### Mating Performance and Singlehood Status (H_2_)

With respect to the multinomial regression analysis, as shown in Table 6, the results indicated a significant main effect of mating performance on relationship status. According to the estimated odds ratios, the produced effect was considerable. For instance, a one-unit increase in mating performance was associated with a 254% increase in the probability of being in a relationship and 232% increase in the probability of being married, than involuntarily single. Apart from Ukraine, this effect was found in all samples.

**Table 6.** Effect of Mating Performance on Relationship Status.

		Relationship status			
	Mating performance	Between relationships single	Voluntarily single	In a relationship	Married
Countries	*p*-Value	OR	OR	OR	OR
Total	<.001	2.53 (<.001)	1.26 (<.001)	3.54 (<.001)	3.32 (<.001)
Austria	<.001	3.18 (<.001)	1.13 (.799)	4.82 (<.001)	5.33 (<.001)
Brazil	<.001	2.72 (<.001)	1.57 (.006)	5.06 (<.001)	5.90 (<.001)
China	<.001	1.23 (.264)	0.801 (.335)	1.81 (.004)	1.05 (.847)
Greece	<.001	1.83 (<.001)	1.01 (.714)	2.42 (<.001)	2.19 (<.001)
Hungary	<.001	2.81 (<.001)	1.21 (.402)	4.81 (<.001)	6.62 (<.001)
Italy	<.001	3.65 (<.001)	2.95 (.39)	9.18 (<.001)	12.65 (<.001)
Japan	<.001	1.90 (.048)	1.76 (.023)	6.64 (<.001)	3.39 (<.001)
Peru	<.001	2.77 (<.010)	1.73 (.017)	4.16 (<.001)	7.32 (<.001)
Poland	<.001	2.39 (.004)	1.73 (0.70)	3.32 (<.001)	3.1 (<.001)
Russia	<.001	2.36 (.005)	0.987 (.966)	3.03 (<.001)	2.76 (<.001)
Spain	<.001	2.74 (.002)	1.38 (.248)	6.12 (<.001)	16.12 (<.001)
Turkey	<.001	1.67 (.004)	0.815 (.150)	2.10 (<.001)	3.18 (<.001)
UK	<.001	3.39 (<.001)	2.04 (.014)	9.28 (<.001)	12.31 (<.001)
Ukraine	.600	1.23 (.689)	0.603 (.489)	1.30 (.641)	1.48 (.355)

*Note*. The reference category was the “Involuntarily single.” The significance of the odds ratios calculated on the basis of the Wald statistic is given in parenthesis.

Repeating the analysis, with voluntary singlehood as the reference category, we found that a one-unit increase in mating performance was associated with an increase in the probability of being between relationships (OR = 2.02, *p *< .001), a decrease in the probability of being involuntarily single (OR = 0.80, *p *< .001), an increase in the probability of being in a relationship (OR = 2.82, *p *< .001), and an increase in the probability of being married (OR = 2.60, *p *< .001), than voluntarily single (the significance of the odds ratios, based on the Wald statistic, is given in parenthesis). Moreover, we repeated the analysis with single-between relationships singlehood as the reference category. The results indicated that a one-unit increase in mating performance was associated with a decrease in the probability to be voluntarily single (OR = 0.50, *p *< .001), a decrease in the probability to be involuntarily single (OR = 0.40, *p *< .001), an increase in the probability to be in a relationship (OR = 1.40, *p *< .001), and an increase in the probability of being married (OR = 1.31, *p *< .001), than between-relationships single. Comparisons between the odds ratios indicated that the effect of mating performance on driving people to be single, versus in a relationship, was higher for the case of involuntary singlehood (OR = 3.42), than voluntary singlehood (OR = 2.82) or between-relationships singlehood (OR = 1.40). Comparisons between the odds ratios indicated that the effect of mating performance on driving people to be single versus married was higher for the case of involuntary singlehood (OR = 3.32), than for voluntary singlehood (OR = 2.60) or between-relationships singlehood (OR = 1.31).

## Discussion

In the current research, we investigated the mating performance and singlehood status in 14 different countries. We found that poor mating performance was relatively common, with about one in four participants indicating low performance, and more than 57% reporting that they faced difficulties in starting and/or keeping a relationship. Men and women did not differ in their mating performance scores, while there was a significant but small age effect, with older participants indicating higher mating performance. Furthermore, poor mating performance was associated with an increased likelihood of singlehood. We also found that nearly 13% of the participants were involuntarily single, with about 15% of the participants indicating that they were voluntarily single, and 10% that they were between relationships. The rates of the three types of singlehood tended to be higher in younger, than in older age groups. Although there was apparent variation, these results were generally consistent across samples.

In our theoretical framework, a considerable discrepancy between the ancestral (where marriages were arranged or mating was forced; see Apostolou, 2007, 2010) and modern mating conditions (where mating is freely exercised) has resulted in several people experiencing poor mating performance, and consequently being involuntarily single. Although our study was not designed to offer a direct test of this hypothesis, our findings are consistent with it, as about one in four participants reported poor mating performance, with the majority indicating that they faced difficulties in starting and/or maintaining an intimate relationship. Also consistent with our original prediction, poor performance was strongly associated with an increased likelihood to be involuntarily single. More specifically, a one-unit increase in mating performance was associated with 254% increased probability of being in an intimate relationship, than involuntarily single. Nevertheless, although about one in four participants indicated poor mating performance, and more than half indicated that they faced difficulties in starting and/or keeping an intimate relationship, the occurrence of involuntary singlehood did not exceed 13%. One interpretation of this finding is that, although many people face poor mating performance, they may overcome their difficulties and manage eventually to attract a partner. One reason is that behavioral adaptations exhibit plasticity, enabling individuals to act in an adaptive way, even if the environment is very different from the one in which these adaptations originally evolved (Mery & Burns, 2010; West-Ebberhard, 2003). To use one example, driving a car is cognitively demanding and evolutionary novel yet, with adequate training, most people manage to drive relatively well. Nevertheless, such plasticity has its limits that, in the mating domain, take the form of many people spending considerable periods of time being involuntarily single.

It should be noted that the occurrence of involuntary singlehood might have been underreported in our sample. One reason is that, to protect their self-esteem, some participants may not have acknowledged that they were single because they faced difficulties attracting a partner and reported instead that they were single because they preferred to be so. Conversely, it could be argued that, because there is some social stigma in being single (DePaulo, 2007), people who preferred to be single may have chosen instead to say that they wanted to be in a relationship, but they could not find appropriate mates. However, the latter is more likely to happen in social interactions, than in anonymous online studies.

A significant effect of age on mating performance was also found, with older indicating a higher mating performance than younger participants. One explanation for this effect is that, as they age, people become more experienced and potentially more effective in dealing with intimate relationships. Nevertheless, the effect was small and was not significant in several countries, suggesting that the experience that comes with age makes little difference in increasing mating performance. More research is required to establish whether more relationship experience translates into higher effectiveness in mating and, if this is the case, how big the effect is.

Consistent with our original prediction, both between-relationships and voluntary singlehood were relatively common. Being voluntarily single was the most frequent form of singlehood, with nearly 15% of the participants indicating that they belonged to this category. One possible explanation is that this category involves people who are single because they either intentionally opt out from the mating market or because they do poorly in it. Consistent with the latter argument, lower mating performance was associated with a higher probability to be between-relationships or voluntarily single, than to be in an intimate relationship.

We also found that all types of singlehood were more frequent in younger than in older age groups. For instance, more than 22% of the participants in the younger age group indicated that they were voluntarily single, versus about 8% in the older age categories. One reason for this finding is that, as people age, they get more relationship experience, which enables them to increase their mating performance and thus reduce the likelihood of being single. However, as discussed above, the effect of age on mating performance was small. Moreover, it has been argued that staying single for a period of time could be beneficial, as it could enable people to build their strengths and subsequently re-enter the mating market with a better chance of success (Apostolou, 2017). Doing so would be more beneficial when young, as they have yet to develop their strengths. Furthermore, as discussed in the introduction, it could be common that people would terminate a relationship to find mates who were a better fit. Such trial-and-error mating should be more frequent in the beginning of one's mating career, as people search for a good match (see also Fisher, 2012). In addition, the mismatch problem affects several mechanisms involved in mating, which usually make it difficult, but not impossible to find mates. Difficult means that it will take longer for individuals to find mates, which, in turn, means that they would be more likely to be single in the early stages of their mating career and less likely to be so at later stages.

Our main findings were relatively consistent across the different countries, but there was also considerable variation. For instance, in all samples, a substantial proportion of people indicated that they experienced poor mating performance, but this proportion was higher in some countries than in others. One source of variation is differences in the demographic characteristics between countries, such as the operational sex ratio (Walter et al., 2021). Another source of variation is the different sampling methods used. For instance, in some cases, data was collected by promoting the study in social media, while in others via the use of a survey research company. Moreover, evolved psychological mechanisms are not rigid but adjust their behavioral output to environmental conditions (Burtăverde & Ene, 2021). To the degree that environmental conditions differ from country to country in domains affecting mating, there would be differences in mating performance and rates of singlehood. For instance, the stability and predictability of the environment can affect mating strategies and thus the rate of singlehood (see Burtăverde & Ene, 2021; Munro et al., 2014; Pisanski & Feinberg, 2013). To use another example, dating applications can potentially give people access to a large pool of available mates, making it easier to find an appropriate one (but see Thomas et al., 2022). In some countries, such applications may be more widely used than in others, which could result in differences in mating performance between them. Cultural factors may also affect how people answer questions related to their mating performance and relationship status. For instance, in more individualistic countries people may tend to exaggerate how well they do in mating. Future studies need to identify how specific sociocultural factors affect mating performance and singlehood status.

Our findings indicate that facing difficulties in the mating domain and being single because of it are not the exception but a common instance, particularly among young age groups. Poor mating performance and singlehood, especially involuntary one, are associated with negative emotions such as loneliness and sadness, as well as with low life-satisfaction (Apostolou et al., 2019c; Costello et al., 2022). It follows that interventions that would enable individuals to improve on their mating performance (e.g., teaching effective flirting skills) and thus decrease the probability to be single, would be of interest to many people. In addition, a wider use of such interventions would potentially have a considerable impact on increasing global happiness and life satisfaction. Furthermore, the relatively high rates of the different types of singlehood strongly suggest that future research in the area needs to distinguish between those who are mated and those are not rather than between those who are married and those who are not (as it is the case today). With respect to those who are not mated, it should further distinguish between those who are voluntarily, involuntarily, or between-relationships so.

### Limitations and Conclusion

The present research has a number of limitations. Firstly, we employed non-probability samples, so our findings may not readily generalize to the population. One possible concern with non-probability samples is the presence of a systematic bias. More specifically, a study of singlehood may attract a disproportionally large number of participants who are single or face difficulties in attracting partners. However, such a systematic bias is unlikely to have affected our results, as our data were part of a larger study on human mating, which was not related to singlehood. In addition, we found significant differences between the samples; yet, these differences may reflect differences in other demographic variables that we did not control, rather than genuine cultural variation.

Moreover, the present study employed self-report instruments, which are subject to several biases, including participants not accurately reporting their mating performance and their singlehood status. In addition, evidence from more countries is necessary to get a better understanding of the cross-cultural occurrence of poor mating performance. Furthermore, the instrument we employed may not fully distinguish between the different singlehood categories. For instance, future research needs to distinguish between those who prefer to be single to work on improving themselves, those who are involuntarily single but say that they are voluntarily so, and those who prefer to be single because they believe that they will fail in the mating market.

Additionally, the present study attempted to understand singlehood within an evolutionary theoretical framework, but other theoretical frameworks could be used for this purpose (see Lahad, 2012). Moving on, the current research aimed to examine mating performance and the different types of singlehood in a post-industrial context and produced evidence, which is consistent with the mismatch argument. However, it was not designed to provide a direct test of this hypothesis. More specifically, we expect that mating performance and singlehood would covary with the degree of parental or familial involvement in mate selection. In particular, *ceteris paribus*, in societies where marriages are arranged, mating performance and the three different types of singlehood would be less common than in societies where marriages are not arranged. Accordingly, future research can test this prediction by including data from pre-industrial societies, where mate choice is regulated.

Moreover, our data are correlational so the nature of the observed associations between mating performance and singlehood status is a matter of interpretation. Our interpretation is that the relationship is causal, going from mating performance to singlehood status: People who, for any reason, are good in attracting and retaining mates (e.g., are good in flirting) would be more likely to be in a relationship than single, compared to those who are not very good in it. This argument possibly explains why many people are single. Another explanation could be that people who are single figure out that they are not good at intimate relationships, so the causal direction is the opposite of the aforementioned. That is, singlehood causes people to report that they have poor mating performance. The problem with this argument is that it does not explain why people are single in the first place. If singlehood was due to a random reason (e.g., a partner died, or one of the partners moved to a distant country and their previous relationship did not last), it is unlikely that they will infer that they are single because they are not good in the mating domain. On the other hand, if people are single because they have certain issues (e.g., shyness or poor flirting skills), they can then infer that their singlehood status is probably due to not doing very well in attracting partners. Yet, in this scenario, singlehood status did not cause them to report low mating performance, but low mating performance caused singlehood. Still, there may be some inverse causality effects. For instance, singles lack the necessary relational occasions to train their mating skills, which can lead them to report poor mating performance.

Although facing difficulties in romantic relationships is common, there is surprising little research examining its occurrence and association with involuntary singlehood. The current study found that poor mating performance was relatively common in the 14 countries studied, and it was strongly associated with increased incidence of singlehood. More studies in different sociocultural settings are necessary to get a better understanding of the reasons why poor mating performance occurs and the different types of singlehood.

## Supplemental Material

sj-docx-1-evp-10.1177_14747049221150169 - Supplemental material for Mating Performance and Singlehood Across 14 Nations Click here for additional data file. Supplemental material, sj-docx-1-evp-10.1177_14747049221150169 for Mating Performance and Singlehood Across 14 Nations by Menelaos Apostolou, Mark Sullman, Béla Birkás, Agata Błachnio, Ekaterina Bushina, Fran Calvo, William Costello, Tanja Dujlovic, Tetiana Hill, Timo Juhani Lajunen, Yanina Lisun, Denisse Manrique-Millones, Oscar Manrique-Pino, Norbert Meskó, Martin Nechtelberger, Yohsuke Ohtsubo, Christian Kenji Ollhoff, Aneta Przepiórka, Ádám Putz, Mariaelena Tagliabue, Burcu Tekeş, Andrew Thomas and Jaroslava Varella Valentova, Marco Antonio Correa Varella, Yan Wang, Paula Wright, Sílvia Font-Mayolas in Evolutionary Psychology

## References

[bibr1-14747049221150169] Adams M. (1976). Single blessedness: Observations on the single status in married society. Basic Books.

[bibr2-14747049221150169] Apostolou M . (2007). Sexual selection under parental choice: The role of parents in the evolution of human mating. Evolution and Human Behavior, 28(6), 403–409. 10.1016/j.evolhumbehav.2007.05.007

[bibr3-14747049221150169] Apostolou M . (2010). Sexual selection under parental choice in agropastoral societies. Evolution and Human Behavior, 31(1), 39–47. 10.1016/j.evolhumbehav.2009.06.010

[bibr4-14747049221150169] ApostolouM . (2012). Sexual selection under parental choice: Evidence from sixteen historical societies. Evolutionary Psychology, 10(3), 504–518. https://doi.org/10.1177%2F14747049120100030822947674

[bibr5-14747049221150169] Apostolou M. (2014). Sexual selection under parental choice: The evolution of human mating behaviour. Psychology Press.

[bibr6-14747049221150169] Apostolou M . (2015). Past, present and why people struggle to establish and maintain intimate relationships. Evolutionary Behavioral Sciences, 9(4), 257–269. 10.1037/ebs0000052

[bibr7-14747049221150169] Apostolou M . (2017). Why people stay single: An evolutionary perspective. Personality and Individual Differences, 111, 263–271. 10.1016/j.paid.2017.02.034

[bibr8-14747049221150169] Apostolou M . (2019). Why men stay single: Evidence from Reddit. Evolutionary Psychological Science, 5(1), 87–97. 10.1007/s40806-018-0163-7

[bibr9-14747049221150169] Apostolou M. Birkás B. da Silva C. S. A. Esposito G. Hsu R. M. C. S. Jonason P. K. Karamanidis K. O. J. Ohtsubo Y. Putz Á. Sznycer D. Thomas A. G. Valentova J. V. Varella M. A. C. Kleisner K. Flegr J. Wang Y . (2022). Reasons of singles for being single: Evidence from Brazil, China, Czech Republic, Greece, Hungary, India, Japan and the UK. Cross-Cultural Research, 55(4), 319–350. 10.1177/10693971211021816

[bibr10-14747049221150169] Apostolou M. Papadopoulou I. Christofi M., Vrontis D . (2019a). Mating performance: Assessing flirting skills, mate signal-detection ability, and shyness effects. Evolutionary Psychology, 17(3), e3. 10.1177/1474704919872416 PMC10299795 31542947

[bibr11-14747049221150169] Apostolou M. Paphiti C. Neza E. Damianou M., Georgiadou P . (2019b). Mating performance: Exploring emotional intelligence, dark triad, jealousy and attachment effects. Journal of Relationships Research, 10, e1. 10.1017/jrr.2018.22

[bibr12-14747049221150169] Apostolou M. Shialos M., Georgiadou P . (2019c). The emotional cost of poor mating performance. Personality and Individual Differences, 138, 188–192. 10.1016/j.paid.2018.10.003

[bibr13-14747049221150169] Apostolou M. Shialos M. Kyrou E. Demetriou A., Papamichael A . (2018). The challenge of starting and keeping a relationship: Prevalence rates and predictors of poor mating performance. Personality and Individual Differences, 122, 19–28. 10.1016/j.paid.2017.10.004

[bibr14-14747049221150169] Apostolou M., Wang Y . (2019). The association between mating performance, marital status, and the length of singlehood: Evidence from Greece and China. Evolutionary Psychology, 17(4). 10.1177/1474704919887706 PMC10480974 31722550

[bibr15-14747049221150169] Broude G. J., Green S. J . (1983). Cross-cultural codes on husband-wife relationships. Ethnology, 22(3), 263–280. 10.2307/3773467

[bibr16-14747049221150169] Burtăverde V. Ene C . (2021). The influence of environmental and social characteristics on women’s mate preferences. Personality and Individual Differences, 175, 110736. 10.1016/j.paid.2021.110736

[bibr17-14747049221150169] Buss D. M . (2009). How can evolutionary psychology successfully explain personality and individual differences? Perspectives on Psychological Science, 4(4), 359–366. 10.1111/j.1745-6924.2009.01138.x 26158983

[bibr18-14747049221150169] Buss D. M. (2016). The evolution of desire: Strategies of human mating (4th ed.). Basic Books.

[bibr19-14747049221150169] Buss D. M . (2017). Sexual conflict in human mating. Current Directions in Psychological Science, 26(4), 307–313. 10.1177/0963721417695559

[bibr20-14747049221150169] Buss D. M . (2020). Evolutionary psychology is a scientific revolution. Evolutionary Behavioral Sciences, 14(4), 316–323. 10.1037/ebs0000210

[bibr21-14747049221150169] Buss D. M. (2021). When men behave badly: The hidden roots of sexual deception, harassment, and assault. Little, Brown Sparks.

[bibr22-14747049221150169] Cherlin A. J. (2009). The marriage-go-round. Alfred A. Knopf.

[bibr23-14747049221150169] Coontz S. (2006). Marriage, a history: How love conquered marriage. Penguin.

[bibr24-14747049221150169] Costello W. Rolon V. Thomas A. G., Schmitt D. (2022). Levels of well-being among men who are incel (Involuntarily Celibate). Evolutionary Psychological Science, 8, 375–390. 10.1007/s40806-022-00336-x

[bibr25-14747049221150169] Crawford C. (1998). Environments and adaptations: Then and now. In Crawford C. Krebs D. L. (Eds.), Handbook of evolutionary psychology (pp. 275–302). Erlbaum.

[bibr26-14747049221150169] DePaulo B. (2007). Singled out: How singles are stereotyped, stigmatized, and ignored, and still live happily ever after. St. Martin’s Press.

[bibr27-14747049221150169] Dinh R. Gildersleve P. Blex C. Yasseri T . (2021). Computational courtship understanding the evolution of online dating through large-scale data analysis. Journal of Computational Social Science, 5(1), 401–426. 10.1007/s42001-021-00132-w

[bibr28-14747049221150169] Ember C. R . (1978). Myths about hunter-gatherers. Ethnology, 17(4), 439–448. 10.2307/3773193

[bibr29-14747049221150169] Fisher H. E. (2012). Serial monogamy and clandestine adultery: Evolution and consequences of the dual human reproductive strategy. In Roberts S. C. (Ed.), Applied evolutionary psychology (pp. 93–111). Oxford University Press.

[bibr30-14747049221150169] Fisher R. A. (1958). The genetic theory of natural selection (2nd ed.). Oxford University Press.

[bibr31-14747049221150169] Goetz C. D. Pillsworth E. G. Buss D. M. Conroy-Beam D . (2019). Evolutionary mismatch in mating. Frontiers in Psychology, 10, 2709. 10.3389/fpsyg.2019.02709 31866904 PMC6904347

[bibr32-14747049221150169] Gutiérrez F. Peri J. M. Baillès E. Sureda B. Gárriz M. Vall G. Cavero M. Mallorqui A. Rodríguez J. R. (2022). A double-track pathway to fast strategy in humans and its personality correlates. Frontiers in Psychology, 13, 889730. https://doi.org/10.3389%2Ffpsyg.2022.889730 35756215 10.3389/fpsyg.2022.889730 PMC9218359

[bibr33-14747049221150169] Keller M. C., Miller G . (2006). Resolving the paradox of common, harmful, heritable mental disorders: Which evolutionary genetic models work best? Behavioral and Brain Sciences, 29(4), 385–404. 10.1017/s0140525×06009095 17094843

[bibr34-14747049221150169] Klinenberg E. (2012). Going solo: The extraordinary rise and surprising appeal of living alone. Duckworth Overlook.

[bibr35-14747049221150169] Lahad K . (2012). Singlehood, waiting, and the sociology of time. Sociological Forum, 27(1), 163–186. 10.1111/j.1573-7861.2011.01306.x

[bibr36-14747049221150169] Lee R. B., Devore I. (1968). Man the hunter. Aldine.

[bibr37-14747049221150169] Li N. P. van Vugt M. Colarelli S. M . (2018). The evolutionary mismatch hypothesis: Implications for psychological science. Current Directions in Psychological Science, 27(1), 38–44. 10.1177/0963721419885877

[bibr38-14747049221150169] Lidborg L. H. Cross C. P. Boothroyd L. G . (2022). A meta-analysis of the association between male dimorphism and fitness outcomes in humans. Elife, 11, e65031. 10.7554/eLife.65031 35179485 PMC9106334

[bibr39-14747049221150169] Međedović J . (2021). Phenotypic signals of sexual selection and fast life history dynamics for the long-term but not short-term mating. Evolutionary Psychology, 19(4), 14747049211057158. 10.1177/14747049211057158 34841944 PMC10461799

[bibr40-14747049221150169] Međedović J . (2022). Long-term mating positively predicts both reproductive fitness and parental investment. Journal of Biosocial Science, 54(5), 912–923. 10.1017/S0021932021000407 34365983

[bibr41-14747049221150169] Mery F., Burns J. G . (2010). Behavioural plasticity: An interaction between evolution and experience. Evolutionary Ecology, 24(3), 571–583. 10.1007/s10682-009-9336-y

[bibr42-14747049221150169] Miller G. (2000). The mating mind. BCA.

[bibr43-14747049221150169] Munro K. R. Flood N. J. McKellar A. E. Reudink M. W . (2014). Female mate preference varies with age and environmental conditions. Behaviour, 151(14), 2059–2081. 10.1163/1568539X-00003231

[bibr44-14747049221150169] Nomaguchi K. M., Bianchi S. M . (2004). Exercise time: Gender differences in the effects of marriage, parenthood, and employment*.* Journal of Marriage and Family, 66(2), 413–430. 10.1111/j.1741-3737.2004.00029.x

[bibr45-14747049221150169] Olejnik S. F. Algina J . (1984). Parametric ANCOVA and the rank transform ANCOVA when the data are conditionally non-normal and heteroscedastic. Journal of Educational Statistics, 9(2), 129–149. 10.3102/10769986009002129

[bibr46-14747049221150169] Pisanski K. Feinberg D. R . (2013). Cross-cultural variation in mate preferences for averageness, symmetry, body size, and masculinity. Cross-Cultural Research, 47(2), 162–197. 10.1177/1069397112471806

[bibr47-14747049221150169] Puts D. A . (2010). Beauty and the beast: Mechanisms of sexual selection in humans. Evolution and Human Behavior, 31(3), 157–175. 10.1016/j.evolhumbehav.2010.02.005

[bibr48-14747049221150169] Puts D. A . (2016). Human sexual selection. Current Opinion in Psychology, 7, 28–32. 10.1016/j.copsyc.2015.07.011

[bibr49-14747049221150169] Sarkisian N. Gerstel N . (2016). Does singlehood isolate or integrate? Examining the link between marital status and ties to kin, friends, and neighbors. Journal of Social and Personal Relationships, 33(3), 361–384. 10.1177/0265407515597564

[bibr50-14747049221150169] Thomas M. F. Binder A. Matthes J . (2022). The agony of partner choice: The effect of excessive partner availability on fear of being single, self-esteem, and partner choice overload. Computers in Human Behavior, 126, 106977. 10.1016/j.chb.2021.106977

[bibr51-14747049221150169] Toma C. L. Hancock J. T . (2010). Looks and lies: The role of physical attractiveness in online dating self-presentation and deception. Communication Research, 37(3), 335–351. https://doi.org/10.1177%2F0093650209356437

[bibr52-14747049221150169] Tooby J., Cosmides L . (1988). The evolution of war and its cognitive foundations. Institute for Evolutionary Studies Technical Report, 88(1), 1–15.

[bibr53-14747049221150169] Trimberger K. E. (2006). The new single women. Beacon Press.

[bibr54-14747049221150169] Walker R. S. Hill K. R. Flinn M. V., Ellsworth R. M . (2011). Evolutionary history of hunter-gatherer marriage practices. PLoS ONE, 6(4), e19066. 10.1371/journal.pone.0019066 21556360 PMC3083418

[bibr55-14747049221150169] Walter K. V. Conroy-Beam D. Buss D. M. Asao K. Sorokowska A. Sorokowski P. , …, & Zupančič M. (2021). Sex differences in human mate preferences vary across sex ratios. Proceedings of the Royal Society B, 288(1955), 20211115. 10.1098/rspb.2021.1115 34284630 PMC8292757

[bibr56-14747049221150169] West-Ebberhard M. J. (2003). Developmental plasticity and evolution. Oxford University Press.

