# Supplementary material for: Mating Performance and Singlehood Across 14 Nations
Source: Evol Psychol. 2023 Jan 3;21(1):14747049221150169. doi: 10.1177/14747049221150169 (PMC10355298; doi:10.1177/14747049221150169)
Supplement: sj-docx-1-evp-10.1177_14747049221150169 - Supplemental material for Mating Performance and Singlehood Across 14 Nations [file sj-docx-1-evp-10.1177_14747049221150169.docx]

Supplementary material A

The frequencies in Table 2 suggest that it was more difficult for people to start than to keep an intimate relationship. In order to examine whether this difference was statistically significant, we performed a series of repeated-measures ANCOVAs, where the difficulty type (i.e., participant’s responses in the question related to the difficulties in starting an intimate relationship in the mating performance instrument versus responses in the question related to keeping an intimate relationship) was entered as a within-subjects categorical independent factor, sex was entered as a between-subjects categorical independent factor, and age as a continuous independent factor. The analysis was repeated 14 times, for each individual and the pooled sample. The UK sample was excluded from this analysis due to an error in the platform used to collect the data, in which participants’ responses to the question “I find it easy to keep a romantic relationship” were not properly recorded. In order to avoid the problem of alpha inflation arising from multiple comparisons, Bonferroni corrections were applied, reducing the alpha level to .004 (.05/14). Thus, the reader may consider any effect above this level not to be significant. The results are presented in Table A, where we can see that, for the pooled sample, there was a significant main effect of difficulty type, with the effect size indicating that it was small. For most samples, the effect was either significant or approached significance, with participants indicating a higher difficulty to start than to keep an intimate relationship. There was one exception, namely Hungary, where the reverse effect was observed.

Table A

*The main effect of difficulty type on mating performance*

| **Countries** |  | *Start a relationship* | *Keep a relationship* |  |  |
| --- | --- | --- | --- | --- | --- |
|  |  | Mean (SD) | Mean (SD) | *p*-value | η_p_^2^ |
| Total |  | 2.68 (1.32) | 2.88 (1.28) | <.001 | .008 |
| Austria |  | 2.90 (1.32) | 3.40 (1.20) | <.001 | .065 |
| Brazil |  | 2.36 (1.42) | 2.49 (1.30) | .016 | .009 |
| China |  | 3.56 (1.19) | 3.60 (1.16) | .394 | .001 |
| Greece |  | 3.04 (1.36) | 3.31 (1.28) | .124 | .004 |
| Hungary |  | 3.42 (1.25) | 2.83 (1.41) | <.001 | .039 |
| Italy |  | 2.50 (1.29) | 2.86 (1.18) | .180 | .004 |
| Japan |  | 2.02 (1.07) | 2.00 (1.07) | .259 | .002 |
| Peru |  | 2.73 (1.25) | 3.08 (1.20) | .001 | .016 |
| Poland |  | 2.92 (1.18) | 3.05 (1.18) | .232 | .003 |
| Russia |  | 2.24 (1.20) | 3.01 (1.25) | .002 | .024 |
| Spain |  | 2.47 (1.36) | 3.02 (1.32) | .001 | .030 |
| Turkey |  | 2.43 (1.13) | 2.66 (1.14) | .019 | .006 |
| Ukraine |  | 2.48 (1.02) | 3.09 (0.94) | .058 | .023 |

Supplementary material B

Table B

*Relationship status across samples for the 18-27 age group*

| **Countries** |  | *Relationship status* | | | | | |
| --- | --- | --- | --- | --- | --- | --- | --- |
|  |  | Between relationships single | Voluntarily single | Involuntarily single | In a relationship | Married | Other |
| Total |  | 12.5 | 23.0 | 16.7 | 41.3 | 3.2 | 3.1 |
| Austria |  | 9.5 | 4.1 | 17.6 | 60.8 | 6.8 | 1.4 |
| Brazil |  | 6.8 | 27.8 | 24.6 | 33.7 | 3.2 | 3.9 |
| China |  | 28.2 | 14.1 | 15.5 | 36.2 | 5.5 | 0.6 |
| Greece |  | 15.3 | 11.3 | 22.1 | 42.9 | 1.5 | 6.7 |
| Hungary |  | 8.2 | 12.9 | 25.0 | 49.3 | 1.8 | 2.9 |
| Italy |  | 11.1 | 11.1 | 17.8 | 52.9 | 0.0 | 7.1 |
| Japan |  | - | - | - | - | - | - |
| Peru |  | 14.2 | 43.2 | 6.3 | 34.7 | 1.4 | 0.2 |
| Poland |  | 13.6 | 4.5 | 9.1 | 40.9 | 31.8 | 0.0 |
| Russia |  | 20.0 | 16.7 | 10.0 | 20.0 | 30.0 | 3.3 |
| Spain |  | 8.9 | 22.1 | 15.0 | 47.9 | 1.4 | 4.7 |
| Turkey |  | 8.6 | 25.9 | 17.2 | 42.3 | 2.8 | 3.3 |
| Ukraine |  | 0.0 | 10.0 | 15.0 | 30.0 | 35.0 | 10 |

*Note.* For the Japanese sample there were very few participants in this age group for meaningful percentages to be estimated.

Supplementary material C

Table C

*Relationship status across samples for the 28-37 age group*

| **Countries** |  | *Relationship status* | | | | | |
| --- | --- | --- | --- | --- | --- | --- | --- |
|  |  | Between relationships single | Voluntarily single | Involuntarily single | In a relationship | Married | Other |
| Total |  | 10.0 | 8.3 | 11.4 | 33.6 | 34.7 | 2.1 |
| Austria |  | 6.6 | 2.0 | 8.6 | 60.9 | 20.5 | 1.3 |
| Brazil |  | 5.3 | 14.6 | 25.8 | 34.4 | 15.2 | 4.6 |
| China |  | 25.8 | 5.9 | 3.8 | 31.7 | 32.8 | 0.0 |
| Greece |  | 14.4 | 12.4 | 18.6 | 29.9 | 22.7 | 2.1 |
| Hungary |  | 5.6 | 4.8 | 18.4 | 33.6 | 33.6 | 4.0 |
| Italy |  | 6.7 | 6.7 | 10.8 | 55.8 | 19.2 | 0.8 |
| Japan |  | 7.5 | 13.8 | 21.3 | 6.3 | 47.5 | 3.8 |
| Peru |  | 12.5 | 25.0 | 2.5 | 52.5 | 7.5 | 0.0 |
| Poland |  | 7.2 | 6.1 | 3.9 | 27.6 | 52.5 | 2.8 |
| Russia |  | 6.8 | 6.2 | 8.2 | 15.1 | 62.3 | 1.4 |
| Spain |  | 9.6 | 13.5 | 11.5 | 44.2 | 19.2 | 1.9 |
| Turkey |  | 8.5 | 11.6 | 10.1 | 28.7 | 38.8 | 2.3 |
| UK |  | 12.8 | 4.6 | 13.8 | 46.8 | 15.6 | 6.4 |
| Ukraine |  | 10.9 | 0.0` | 4.3 | 13.0 | 71.7 | 0.0 |

Supplementary material D

Table D

*Relationship status across samples for the 38< age group*

| **Countries** |  | *Relationship status* | | | | | |
| --- | --- | --- | --- | --- | --- | --- | --- |
|  |  | Between relationships single | Voluntarily single | Involuntarily single | In a relationship | Married | Other |
| Total |  | 6.0 | 8.6 | 8.0 | 13.9 | 58.8 | 4.6 |
| Austria |  | 7.1 | 3.2 | 6.3 | 31.0 | 48.4 | 4.0 |
| Brazil |  | 7.5 | 13.8 | 12.1 | 19.5 | 44.3 | 2.9 |
| China |  | - | - | - | - | - | - |
| Greece |  | 11.5 | 6.2 | 8.8 | 16.8 | 47.8 | 8.8 |
| Hungary |  | 8.4 | 1.1 | 11.6 | 30.5 | 43.2 | 5.3 |
| Italy |  | 3.3 | 2.4 | 3.3 | 26.8 | 60.2 | 4.1 |
| Japan |  | 3.6 | 11.7 | 8.3 | 2.8 | 67.8 | 5.9 |
| Peru |  | - | - | - | - | - | - |
| Poland |  | 5.8 | 7.1 | 5.2 | 19.7 | 59.7 | 2.6 |
| Russia |  | 4.0 | 7.6 | 10.7 | 16.5 | 59.4 | 1.8 |
| Spain |  | 4.5 | 12.5 | 8.0 | 9.1 | 55.7 | 10.2 |
| Turkey |  | 4.0 | 9.9 | 2.0 | 5.9 | 71.3 | 6.9 |
| UK |  | 8.5 | 17.1 | 9.8 | 19.5 | 42.7 | 2.4 |
| Ukraine |  | 20.4 | 7.5 | 12.9 | 4.3 | 52.7 | 2.2 |

*Note.* For the Chinese and the Peruvian samples there were very few participants in this age group for meaningful percentages to be estimated.

Supplementary material E

Table E

*Significant differences between the samples in relationship status*.

| **Relationship status** | *Sample* | | | | | | | | | | | | | |
| --- | --- | --- | --- | --- | --- | --- | --- | --- | --- | --- | --- | --- | --- | --- |
|  | Greece | Turkey | China | Austria | Brazil | Italy | Russia | Ukraine | Poland | Spain | Hungary | Japan | Peru | UK |
|  | Count | Count | Count | Count | Count | Count | Count | Count | Count | Count | Count | Count | Count | Count |
| Between relationships single | 80_a_ | 77_b,d,e_ | 146_c_ | 29_a,b,d_ | 44_b,d,e_ | 37_a,b,d_ | 25_b,d,e_ | 24_a,b,c_ | 34_b,d,e_ | 29_a,b,d_ | 38_a,b,d_ | 28_d_ | 87_a_ | 40_a,e_ |
| Voluntarily single | 59_a,d,e,i_ | 212_b_ | 60_a,d,e,i_ | 11_c_ | 133_b_ | 36_a,c,i_ | 31_a,c,i_ | 9_a,c,i_ | 34_a,c_ | 65_b,d,f_ | 43_c,e,g,i_ | 85_a,f,g,i_ | 257_h_ | 54_b,i_ |
| Involuntarily single | 107_a,e,f,i,m_ | 139_a,b,c,j_ | 61_c,l_ | 36_c,d,h_ | 141_e_ | 57_b,c,f_ | 39_c,g,h_ | 17_a,c,e,h_ | 25_h_ | 45_b,c,i,j_ | 104_e,j,m_ | 70_c,h,k_ | 38_h,l,n_ | 44_b,c,m,n_ |
| In a relationship | 195_a,f,i_ | 349_a,f,i_ | 185_a,f,i_ | 184_b_ | 192_a,d_ | 219_b_ | 66_c,e_ | 16_c,g_ | 120_d,e_ | 133_a,b_ | 209_b,f,h_ | 26_g_ | 222_a,h,i_ | 154_b,i_ |
| Married | 86_a_ | 142_a_ | 83_a_ | 103_b_ | 112_a_ | 97_a,b_ | 234_c_ | 89_c_ | 287_c_ | 63_a,b_ | 88_a_ | 458_c_ | 21_d_ | 52_a_ |
| Other | 36_a_ | 34_a_ | 2_b,c_ | 10_a,b_ | 24_a_ | 22_a_ | 7_a,b,c_ | 4_a,b,c_ | 13_a,b_ | 20_a_ | 18_a_ | 39_a_ | 1_c_ | 16_a_ |

*Note*. Values in the same row and sub-table not sharing the same subscript are significantly different at *p*< .05 in the two-sided test of equality for column proportions. Cells with no subscript are not included in the test. Tests are adjusted for all pairwise comparisons within a row of each innermost sub-table using the Bonferroni correction.

*Note 2*. The Chi-square was 2319.6 (*p* <. 001).

Supplementary material F

Table F

*Significant differences in relationship status across age groups*

| **Relationship status** | *Age* | | |
| --- | --- | --- | --- |
|  | 18-27 | 28-37 | 38< |
|  | Count | Count | Count |
| Between relationships single | 395_a_ | 150_b_ | 124_c_ |
| Voluntarily single | 726_a_ | 125_b_ | 179_b_ |
| Involuntarily single | 527_a_ | 171_b_ | 167_c_ |
| In a relationship | 1303_a_ | 505_b_ | 290_c_ |
| Married | 102_a_ | 522_b_ | 1224_c_ |
| Other | 99_a_ | 31_a_ | 96_b_ |

*Note*. Values in the same row and sub-table not sharing the same subscript are significantly different at *p*< .05 in the two-sided test of equality for column proportions. Cells with no subscript are not included in the test. Tests are adjusted for all pairwise comparisons within a row of each innermost sub-table using the Bonferroni correction.

*Note 2*. The Chi-square was 2136.9 (*p* <. 001).

Supplementary material G

In Table G, we estimated the mean length of the singlehood spell (i.e., how many years participants indicated that, at the time of study, they were single) for the sub-sample of eight societies. Across the whole sample, people spent on average 5.73 years (*SD* = 8.23) single. In order to examine whether mating performance predicted the length of singlehood spells, we run a series of ANCOVAs, where the years being single was entered as the dependent variable, sex and sample were entered as categorical independent variables, and age and mating performance were entered as continuous independent variables. Overall, nine such tests were performed (i.e., one for the pooled sample and nine for the individual samples), so the alpha level was set to .006 (.05/9). As we can see from Table G, in most cases there was a significant main effect of mating performance, with a negative coefficient, indicating that higher scores in mating performance were associated with fewer years of singlehood. We can also see that there were significant differences between the samples.

We also calculated, for the pooled sample, the number of years individuals were in their current relationship status, which included: involuntary single 7.60 (*SD* = 7.96) years, voluntarily single 5.58 (*SD* = 7.86) years, and participants who were between relationships 1.64 (*SD* = 3.44) years. In order to examine whether these differences were significant, we performed an ANCOVA test where the years single was the dependent variable, type of singlehood and sex were the categorical independent variables, and age was the continuous variable. The Bonferroni post-hoc tests indicated that these means were significantly different from each other. In addition, we wanted to examine whether mating performance predicted years single, for each type of singlehood. Accordingly, we run an ANCOVA test where years single was the dependent variable, sex was the categorical independent variable, and mating performance and age were the continuous independent variables. The analysis was performed three times, once for each category of singlehood. For the between-relationships case, no significant main effect of mating performance was found (*p* = .201). For the voluntary singlehood case, a significant main effect of mating performance was found [*F*(1, 466) = 25.34, *p* < .001 η_p_^2^ = .052], with a negative coefficient (-2.08). Similarly, for the involuntary singlehood case, a significant main effect of mating performance was found [*F*(1, 318) = 27.72, *p* < .001 η_p_^2^ = .080], with a negative coefficient (-3.01).

Table G

*The effect of mating performance on the length of singlehood*

| **Countries** |  | *Mean (SD)* | *Mating performance** | | *Sex* | | *Age* | | *Sample*** |
| --- | --- | --- | --- | --- | --- | --- | --- | --- | --- |
|  |  |  | *p*-value | η_p_^2^ | *p*-value | η_p_^2^ | *p*-value | η_p_^2^ | *p*-value*** |
| Total |  | 5.73 (8.23) | <.001 | .023 | .057 | .002 | <.001 | .152 | <.001 |
| Austria |  | 4.12 (6.16) | <.001 | .192 | .178 | .028 | .821 | .001 | I, R, U |
| Brazil |  | 7.49 (9.09) | <.001 | .104 | .694 | .001 | .490 | .002 | I, R, U |
| Greece |  | 7.00 (8.88) | .236 | .005 | .013 | .024 | <.001 | .633 | A, I, R, U |
| Italy |  | 5.01 (7.54) | <.001 | .153 | .006 | .061 | .778 | .001 | G, T, B, Pe |
| Peru |  | 4.62 (6.07) | .002 | .028 | .611 | .001 | .346 | .003 | A, I, R, U |
| Russia |  | 5.09 (7.63) | <.001 | .140 | .876 | .000 | .003 | .097 | G, T, B, Pe |
| Turkey |  | 5.34 (8.96) | .089 | .006 | .624 | .000 | <.001 | .500 | I, R, U |
| Ukraine |  | 4.91 (6.12) | .001 | .212 | .002 | .205 | .498 | .011 | G, T, B, Pe |

* In all instances where mating performance was significant, the regression coefficient was negative.

** In this column the result of the Bonferroni post-hoc test are presented. For each country in the raw, the initials of the countries for which there was a significant difference in the mean mating performance scores are presented. Note further that ‘P’ refers to Poland and ‘Pe’ to Peru.

*** The η_p_^2^ for the sample variable was .041.
